# Effects of Sleep Duration on Electroencephalographic and Autonomic Nervous System Responses to High-Intensity Exercise

**DOI:** 10.3390/healthcare14060728

**Published:** 2026-03-12

**Authors:** Jae-Hyun Jung, Wi-Young So, Jae-Myun Ko

**Affiliations:** 1 Department of Physical Education, Sookmyung Women’s University, Seoul 04310, Republic of Korea; jhjung0823@gmail.com; 2 Department of Sports Medicine, College of Humanities, Korea National University of Transportation, Chungju-si 27469, Republic of Korea; 3 Department of Physical Education, Yonsei University, Seoul 03722, Republic of Korea

**Keywords:** autonomic nervous system, electroencephalography, heart rate variability, high-intensity exercise, sleep duration

## Abstract

**Objective**: This study examined whether changes in electroencephalography (EEG)-derived indices, photoplethysmography (PPG)-derived autonomic nervous system indices, heart rate, and rating of perceived exertion (RPE) post-high-intensity exercise differ depending on sleep duration. **Methods**: Forty physically healthy female university students in their twenties were randomly assigned to the sleep restriction (SR) or normal sleep (NS) group. EEG-derived indices—the theta-to-beta ratio (TBR) and spectral edge frequency at 90% (SEF-90)—and PPG-derived autonomic nervous system indices (HRV index, sympathetic activity, and parasympathetic activity) were measured for one minute at rest before exercise and for one minute immediately after exercise. Heart rate was assessed at rest, immediately after exercise, and at 5, 10, and 15 min post-exercise. The group × time interaction effects were assessed using two-way mixed-design analysis of variance, followed by post hoc analyses. **Results:** TBR increased significantly post-exercise in the SR group (*p* = 0.002) with no significant change in the NS group. SEF-90 decreased significantly in the SR group (*p* < 0.001) with no significant change in the NS group. The HRV index decreased significantly in the SR group (*p* = 0.004) with no significant change in the NS group. Sympathetic activity increased and parasympathetic activity decreased significantly in the SR group (both *p* < 0.001). Heart rate was significantly higher in the SR group at rest (*p* < 0.001), immediately after exercise (*p* = 0.020), and 5 min post-exercise (*p* = 0.009). RPE was significantly higher in the SR group (*p* = 0.003). **Conclusions:** In healthy young adult women, the central and autonomic nervous systems respond differently to high-intensity exercise depending on sleep duration.

## 1. Introduction

Sleep is one of the most fundamental processes in physical and psychological recovery and is involved in core functions such as cognitive performance, immune regulation, metabolic balance, and emotional control. In particular, the human brain performs critical processes during sleep, such as information integration, memory consolidation, and emotional regulation, all of which are essential for maintaining healthy functioning and optimal performance [1,2,3]. However, modern living environments are replete with factors that disrupt natural sleep. University students frequently experience reduced sleep duration and impaired sleep quality due to excessive academic workload, overuse of digital devices, and lifestyle habits, such as caffeine or alcohol consumption. This places the population at a heightened risk of intentional or unintentional sleep deprivation.

Recent studies have reported that smartphone addiction is closely associated with decreased sleep duration and poorer sleep quality among university students, and this relationship is further intensified by depressive symptoms [4,5]. Sleep insufficiency exerts direct effects on the central nervous system, leading to delayed behavioral responses, cognitive impairment, and emotional instability [6,7]. In particular, impaired prefrontal cortical function has been linked to reduced attention, working memory, reaction speed, and decision-making capacity [8,9]. Repeated exposure to such deficits may cause long-term deterioration of neural function.

In addition to central effects, sleep deprivation also affects autonomic regulation. Sleep loss induces hyperactivation of the sympathetic nervous system, resulting in elevated heart rate, increased blood pressure, and heightened cortisol levels [10,11].

Sleep insufficiency is also detrimental to exercise performance. Under conditions of inadequate sleep, reductions in endurance capacity, reaction speed, and post-exercise heart rate recovery have been reported [12,13,14]. These physiological alterations are accompanied by increased ratings of perceived exertion (RPE). Even when exercise intensity remains constant, individuals experiencing sleep loss tend to report higher RPE values, a phenomenon often interpreted as central fatigue [15,16].

In recent years, electroencephalography (EEG) has been increasingly utilized to quantitatively examine the effects of sleep duration. EEG, a noninvasive technique, measures neural activity in the brain through electrical signals and is considered a meaningful physiological indicator of arousal, attentional engagement, and cognitive fatigue. Under conditions of sleep deprivation (or sleep restriction), there are alterations in EEG frequency components, including low-frequency bands such as delta (δ, 0.5–4 Hz) and theta (θ, 4–8 Hz), and these changes indicate accumulated fatigue and reduced levels of arousal [17].

Examining EEG outcomes post-exercise helps elucidate how the nervous system responds to exercise stimuli. For example, EEG alterations immediately following high-intensity exercise tend to reflect the activation of the sensorimotor integration required for exercise performance; however, such alterations may be attenuated or delayed under conditions of sleep deprivation.

Therefore, comparing EEG responses under different sleep conditions provides important insights into the neurophysiological mechanisms underlying fatigue and recovery. Previous research on sleep loss has distinguished between total sleep deprivation and partial sleep restriction. Total sleep deprivation involves the complete absence of sleep for a defined period, whereas partial sleep restriction refers to a reduction in habitual sleep duration and is often considered a more ecologically valid and ethically feasible experimental model. Prior studies employing partial sleep restriction have reported alterations in EEG indices and cognitive impairments under conditions of experimentally reduced sleep duration [18,19]. In addition, Van Dongen et al. [20] directly compared chronic partial sleep restriction (e.g., restricted time in bed across consecutive days) with total sleep deprivation, demonstrating that partial restriction can produce cumulative, dose-dependent neurobehavioral deficits over time.

Despite this, few studies have examined how sleep duration affects changes in EEG outcomes and subjective fatigue post-exercise, and experimental investigations focusing on university students remain scarce. Furthermore, Glavin et al. [21] reported that sleep, exercise, and mood interact differently depending on sex, with young adult women being more susceptible than men to the effects of altered sleep and exercise on mood. Additionally, Hajali et al. [22] suggested that, due to the influence of sex hormones, sleep loss-induced cognitive impairments may be more sensitive and pronounced in women.

Accordingly, women exhibit relatively greater neurophysiological and autonomic sensitivity to sleep restriction due to sex-related differences in hormonal regulation, stress reactivity, and sleep architecture [21,22]. This provides a theoretical rationale for focusing on a female sample in the present study.

Therefore, this study examined whether the response of the central nervous system to high-intensity exercise differs based on sleep duration. In particular, it analyzed whether changes in EEG outcomes post-high-intensity exercise differ under the conditions of sleep restriction and normal sleep among healthy women in their twenties. RPE and baseline physical fitness factors (muscular strength, flexibility, and balance) were evaluated as supplementary indicators. The findings of this study may enhance physiological understanding of the interaction between sleep and high-intensity exercise performance and provide foundational data for developing strategies to manage exercise-related fatigue in young adult women.

The primary hypothesis of this study was that short-term sleep restriction would exacerbate exercise-induced alterations in central nervous system activity, as reflected by changes in TBR and SEF-90, compared with normal sleep. The secondary hypothesis was that sleep restriction would lead to greater autonomic imbalance and cardiovascular strain following exercise, as evidenced by altered HRV-related indices, heart rate responses, and RPE.

## 2. Materials and Methods

### 2.1. Participants

This study recruited physically healthy female university students in their twenties. The inclusion criteria were: (1) being physically healthy with no diagnosed medical conditions or factors that could contraindicate high-intensity exercise; (2) having a regular menstrual cycle and no history of hormone medication use within the last three months; (3) being able to comply fully with the study conditions throughout the experimental period; and (4) providing voluntary written informed consent after receiving a sufficient explanation of the study purpose and procedures. The exclusion criteria were: (1) being diagnosed with a neurological or cardiovascular disorder; (2) being pregnant or the possibility of pregnancy during the experimental period; (3) having any medical contraindications to high-intensity exercise; and (4) having resting blood pressure or heart rate values outside the normal ranges. (5) being in the menstrual phase at the time of testing. Although participants in the active menstrual bleeding phase were excluded, the specific phase of the menstrual cycle (e.g., follicular or luteal phase) was not further standardized or hormonally verified at the time of testing. Participants were tested irrespective of their cycle phase, and no hormonal assays were performed. Data were collected in October 2025.

We estimated the sample size using G*power software (G*power 3.1.9.7, Heinrich-Heine-University, Düsseldorf, Germany). For independent *t*-tests comparing mean differences between two groups, with a significance level (α) of 0.05, a statistical power of 0.80, and a minimum effect size (Cohen’s d) of 0.91, the minimum sample size was 20 participants per group. Accordingly, we randomly assigned 20 participants each to either a sleep restriction (SR) group (*n* = 20) or a normal sleep (NS) group (*n* = 20) using a computer-generated random number sequence (Microsoft Excel, Microsoft Corp., Redmond, WA, USA). The randomization procedure was performed by a researcher not involved in outcome assessment. All participants were screened to confirm eligibility according to the inclusion and exclusion criteria, and all provided written informed consent using their handwritten signature after receiving a sufficient explanation of the study purpose and procedures. This study was conducted after obtaining approval from the Institutional Review Board of Sookmyung Women’s University, Seoul, Republic of Korea (IRB No. SMWU-2506-HR-041-01; Approval date: 19 September 2025). Table 1 presents the characteristics of the participants. 

### 2.2. Sleep Conditions for the Sleep Restriction and Normal Sleep Groups

**Figure 1:**
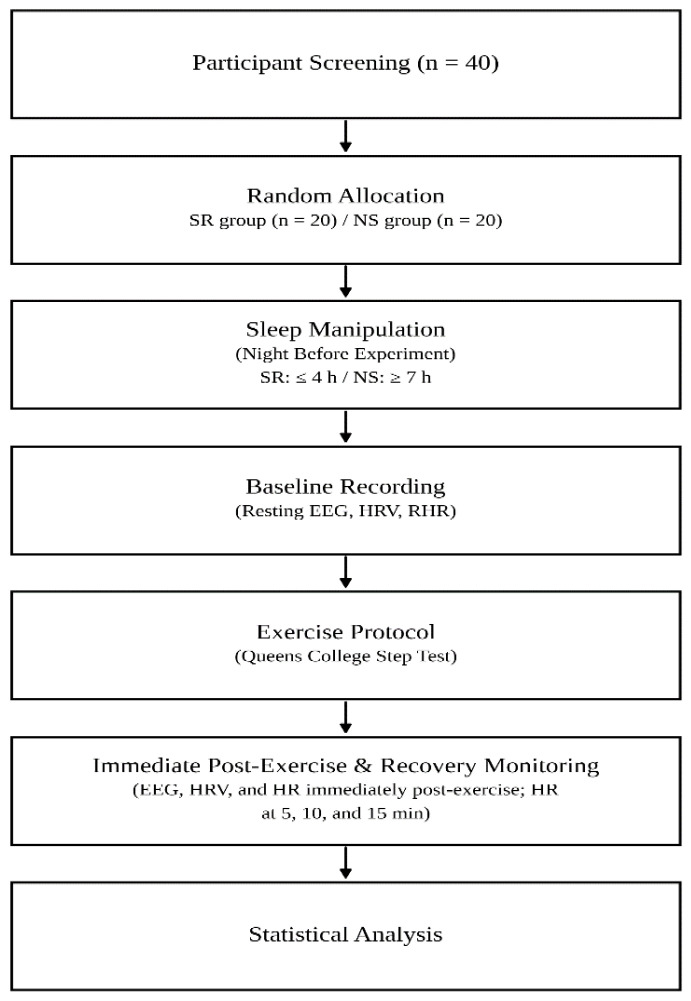
Experimental Procedure.

Figure 1 presents the experimental procedure. The NS group was instructed to maintain their usual sleep habits and obtain approximately eight hours of sleep on the night before the experiment. In contrast, the SR group was instructed to restrict total sleep duration to approximately four hours on the night preceding the experiment. This sleep-restriction protocol was based on prior experimental studies demonstrating that partial sleep restriction of 3–5 h of time in bed is sufficient to induce measurable neurobehavioral and physiological alterations [18,19,20]. Accordingly, a target of approximately four hours was selected as a moderate and experimentally validated level of partial sleep restriction that balances ecological relevance and participant safety.

In addition, participants were instructed to refrain from caffeine and alcohol consumption and to avoid vigorous physical activity for at least 24 h prior to the experiment to minimize potential confounding effects. Participants were also instructed to avoid daytime naps during the sleep manipulation period.

Sleep compliance was not verified using objective sleep-monitoring devices such as actigraphy or polysomnography; instead, sleep duration was assessed using self-reported sleep diaries. Bedtime, wake-up time, and time in bed (TIB) were recorded by the participants in the sleep diary, and, when necessary, adherence was cross-checked using time-stamped electronic communication records. Although practical for field-based experimentation, reliance on self-reported sleep diaries may introduce reporting bias and limit objective verification of sleep duration.

The sleep restriction condition was applied after obtaining voluntary consent from the participants. Those who failed to comply with the prescribed sleep duration were either excluded from the experiment or had their experimental session rescheduled. The mean self-reported sleep duration was 7.90 ± 1.07 h in the NS group and 3.65 ± 1.08 h in the SR group (Table 1). All participants included in the final analysis met the predefined sleep duration criteria. On the day of the experiment, participants’ subjective sleepiness was assessed using the Stanford Sleepiness Scale (SSS). This scale evaluates current subjective sleepiness on a 7-point scale ranging from 1 to 7. A score of five or higher was considered indicative of excessive sleepiness, and in such cases, the experiment was postponed or terminated to ensure participant safety. The SSS has been widely used in sleep restriction research and has demonstrated good reliability and validity [23,24].

### 2.3. Baseline Assessments (Measurement of Body Composition and Physical Fitness)

Baseline assessments consisted of anthropometric measurements and evaluations of basic physical fitness. All participants underwent measurements of height (cm) and body weight (kg) using a body composition analyzer (BSM330, Biospace, Seoul, Republic of Korea). Body composition parameters, including body fat percentage (%), were assessed using a bioelectrical impedance analysis device (InBody 720, Biospace, Seoul, Republic of Korea). To minimize measurement error, participants were asked to maintain a fasting state for at least 12 h prior to assessment and void bowel and bladder immediately before the measurements. In addition, all metallic items that could affect electrical resistance (e.g., watches and rings) were removed, and measurements were conducted using standardized postures to ensure accuracy.

For the assessment of basic physical fitness, measurements were conducted according to each sleep condition group, respectively. Muscular strength was evaluated by measuring upper-limb strength using a digital handgrip dynamometer (T.K.K. 5401, Tokyo, Japan). Before the measurement, the dynamometer was adjusted to fit the participant’s hand. Next, the participants were instructed to grip the dynamometer as forcefully as possible for three seconds with the elbow naturally extended. Each hand was tested twice, and the highest value was recorded for analysis. Flexibility was assessed using a flexibility measurement device (T.K.K. 5403, Tokyo, Japan). Participants sat with both feet placed vertically against the footplate of the device and, with both hands overlapped, slowly bent the trunk forward to push the scale with their fingertips. The maximum reach distance (cm) was recorded. Two trials were performed, and the highest value was used for analysis. The examiner ensured that the fingertips did not bend and that no bouncing movements were performed during the measurement. Balance was assessed using a digital stopwatch (HS-80TW, Casio^®^, Tokyo, Japan). The participants stood with arms relaxed at the sides and maintained a single-leg stance with the lifted knee flexed at 90°. This test was performed with the eyes closed, and the trial was terminated when the contralateral foot touched the ground or when postural stability was lost. Each leg was tested twice, and the highest value was used for analysis.

### 2.4. EEG-Derived Indices and Photoplethysmography-Derived Autonomic Nervous System Indices

The EEG indices analyzed in this study were the theta-to-beta ratio (TBR, θ/β ratio) and spectral edge frequency at 90% (SEF-90). The photoplethysmography (PPG)-derived autonomic nervous system indices included the heart rate variability (HRV) index, sympathetic activity, and parasympathetic activity.

After resting for at least 10 min prior to exercise, participants were seated and fitted with a portable integrated device (Omnifit Mindcare, Seoul, Republic of Korea) capable of simultaneous EEG and PPG acquisition. EEG and PPG signals were recorded simultaneously for one minute under eyes-closed resting conditions. Immediately following high-intensity exercise, measurements were repeated for one minute under identical posture and environmental conditions. All measurements were conducted in a quiet, temperature-controlled laboratory environment maintained at 22–24 °C to minimize environmental influences on autonomic and electrophysiological parameters.

EEG signals were recorded from the prefrontal region using a two-channel configuration, with electrodes placed at Fp1 and Fp2 according to the international 10–20 system. This configuration was selected to focus on prefrontal activity related to arousal and attentional regulation; however, it does not capture activity from sensorimotor or other cortical regions. The reference and ground electrodes were attached via an earlobe clip, which also housed the integrated PPG sensor.

The EEG sampling rate and band-pass filtering parameters followed the manufacturer’s predefined technical specifications. EEG signals were sampled at 250 Hz and recorded within a 3–41 Hz band-pass range. Impedance thresholds followed the manufacturer’s default device settings. No user-defined filtering or offline reprocessing was performed in this study. Artifact detection and signal stabilization were managed using the device’s built-in automated signal processing algorithms.

TBR was calculated as the ratio of relative power in the theta band (4–8 Hz) to the beta band (13–30 Hz). SEF-90 was defined as the frequency below which 90% of the cumulative EEG power spectrum was contained.

PPG signals were obtained from peripheral vascular photo-reflective measurements, and heart rate (HR) and HRV-related indices were derived using the device’s embedded algorithms. PPG-based HRV analysis was automatically computed using the embedded algorithms of the device. According to the manufacturer’s technical documentation, the HRV index represents a composite measure derived from beat-to-beat variability extracted from PPG signals and is calculated based on time-domain and/or frequency-domain HRV parameters. The reported “sympathetic activity” and “parasympathetic activity” indices are algorithm-derived estimates based on HRV-related variables and do not represent direct physiological measurements of autonomic nerve activity. The detailed internal computational procedures of the embedded algorithms are proprietary and not fully disclosed by the manufacturer. All device setup procedures, signal acquisition, and experimental protocols were performed by trained research personnel following standardized operational guidelines.

Although standard short-term HRV assessment typically recommends approximately five minutes of recording, prior studies have demonstrated that ultra-short-term recordings (e.g., 60 s), particularly for time-domain indices under resting conditions, can provide acceptable agreement with conventional short-term recordings. Therefore, the one-minute recording duration used in this study was considered appropriate for evaluating resting autonomic responses. However, frequency-domain interpretations were approached cautiously. Given the proprietary nature of the embedded algorithms, full methodological transparency of the HRV computation process is limited, and therefore, the findings should be interpreted with consideration of this constraint.

### 2.5. High-Intensity Exercise Protocol

Exercise was performed according to the standard protocol of the Queens College Step Test (QCT), which is commonly classified as a submaximal step test for indirectly estimating cardiorespiratory fitness (VO_2_max). The protocol was conducted using a step height of 33 cm for women, a duration of 3 min, and a stepping rate of 22 steps per minute. Participants stepped up and down the platform in an alternating-leg pattern, and a smartphone-based metronome was used to standardize cadence.

Prior to data collection, participants were familiarized with the stepping procedure through standardized instructions and brief practice trials to minimize potential learning effects. Although the QCT is traditionally considered a submaximal test for estimating cardiorespiratory fitness, the present protocol was implemented as a standardized stepping stimulus intended to induce a robust acute physiological response.

Heart rate (HR) was measured at rest, immediately after exercise, and at 5, 10, and 15 min post-exercise using a Polar heart rate monitor in accordance with the standard QCT procedure. Heart rate was assessed at predefined recovery time points to ensure standardized comparisons between groups. Although continuous monitoring may provide higher temporal resolution, the selected time points allowed for consistent evaluation of recovery trends across the early and mid-recovery phases. In the present sample, peak HR during the stepping protocol reached approximately 90% of age-predicted HRmax. Immediately after completion of exercise, rating of perceived exertion (RPE) was assessed using the Borg 6–20 scale, and mean RPE values were ≥17 [25]. According to established exercise intensity classifications, these values correspond to vigorous intensity.

### 2.6. Data Analysis

Descriptive statistics were calculated for all variables and presented as means and standard deviations. Normality of the data was assessed using the Shapiro–Wilk test, and homogeneity of variances was examined using Levene’s test. For repeated-measures factors, sphericity was evaluated using Mauchly’s test, and Greenhouse–Geisser corrections were applied when the assumption of sphericity was violated.

To determine whether changes in EEG-derived indices and PPG-derived autonomic nervous system indices post-exercise differed depending on sleep duration, a two-way mixed analysis of variance (ANOVA; group × time) was performed. When significant interaction effects were observed, paired *t*-tests and independent *t*-tests were conducted to determine the simple main effects of each independent variable.

In addition, to address potential baseline imbalances observed in certain variables (e.g., SEF-90 and resting heart rate), supplementary robustness analyses were conducted using pre–post change scores (Δ = post − pre). Between-group comparisons of these change scores were performed using independent-sample *t*-tests to examine whether the observed response patterns remained consistent when accounting for baseline differences.

HR and RPE between the two groups were compared using independent-sample *t*-tests. Similarly, between-group differences in baseline physical fitness were assessed using independent-sample *t*-tests.

The primary outcome variables were predefined based on theoretical considerations. Specifically, EEG-derived indices (TBR and SEF-90) were defined as primary outcomes, whereas HRV-related indices, heart rate measures, and RPE were considered secondary outcomes. Given that each variable represents a distinct physiological construct, no formal correction for multiple comparisons was applied. Although no formal adjustment for multiple comparisons was applied, the presence of multiple outcome variables may increase the risk of Type I error; therefore, statistically significant findings were interpreted with caution. Effect sizes (partial η^2^) were reported alongside *p*-values to provide a more comprehensive interpretation of the findings. All analyses were conducted using SPSS Statistics 25 (IBM Corp., Armonk, NY, USA), and statistical significance was set at α = 0.05.

## 3. Results

### 3.1. Changes in EEG-Derived Indices Post-High-Intensity Exercise in the SR and NS Groups

**Figure 2:**
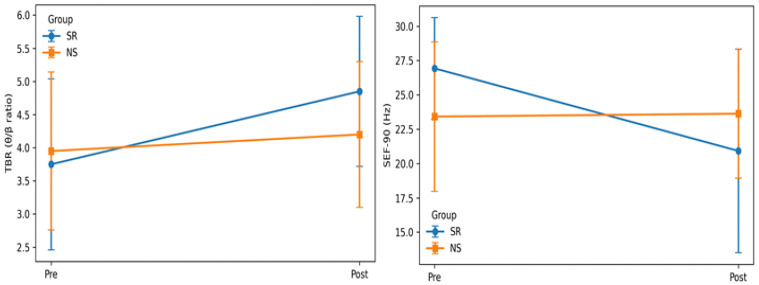
EEG responses to high-intensity exercise under sleep restriction and normal sleep.

A statistically significant group × time interaction effect was observed for TBR [F (1, 38) = 4.188, *p* = 0.048, partial η^2^ = 0.099]. In addition, a significant main effect of time was observed [F (1, 38) = 10.565, *p* = 0.002, partial η^2^ = 0.218] (Table 2 and Figure 2). Post hoc analyses using paired-sample *t*-tests revealed a significant pre–post difference in TBR in the SR group (*p* = 0.002), whereas no significant difference was observed in the NS group (*p* = 0.398). Independent-sample *t*-tests conducted between groups showed no significant between-group differences at either the pre-exercise (*p* = 0.614) or post-exercise (*p* = 0.075) time point. Regarding SEF-90, a significant group × time interaction effect was observed [F (1, 38) = 13.472, *p* = 0.001, partial η^2^ = 0.262]. A significant main effect of time was also found [F (1, 38) = 11.714, *p* = 0.001, partial η^2^ = 0.236]. Post hoc paired-sample *t*-tests revealed a significant pre–post difference in SEF-90 in the SR group (*p* < 0.001), whereas no significant difference was observed in the NS group (*p* = 0.826). Independent-sample *t*-tests comparing the two groups showed a significant between-group difference at the pre-exercise time point (*p* = 0.022) but no significant difference at the post-exercise time point (*p* = 0.175). Accordingly, the group × time effect for SEF-90 should be interpreted primarily as reflecting differential pre–post change trajectories rather than absolute between-group post-exercise differences, given the significant between-group difference at baseline.

Additional analyses based on change scores (ΔSEF-90) further demonstrated that the magnitude of pre–post reduction was significantly greater in the SR group than in the NS group, supporting the interpretation that the observed interaction effect reflects differential change trajectories across sleep conditions.

### 3.2. Changes in PPG-Derived Autonomic Nervous System Indices Post-High-Intensity Exercise in the SR and NS Groups

**Figure 3:**
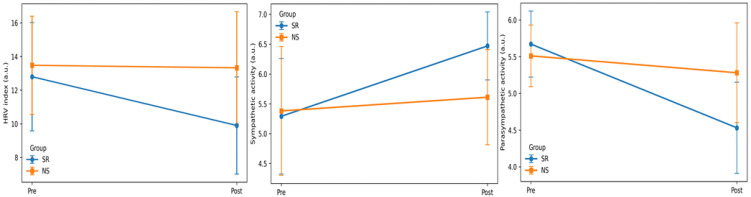
Autonomic responses to high-intensity exercise under sleep restriction and normal sleep.

A significant group × time interaction effect was observed for the HRV index [F (1, 38) = 4.513, *p* = 0.040, partial η^2^ = 0.106]. A significant main effect of time was also observed [F (1, 38) = 5.574, *p* = 0.023, partial η^2^ = 0.128] (Table 3 and Figure 3). Post hoc paired-sample *t*-tests revealed a significant pre–post difference in the SR group (*p* = 0.004), whereas no significant difference was observed in the NS group (*p* = 0.871). Independent-sample *t*-tests comparing the two groups indicated no significant between-group difference at the pre-exercise time point (*p* = 0.488), whereas a significant between-group difference was observed at the post-exercise time point (*p* = 0.001). Regarding sympathetic activity, a significant group × time interaction effect was observed [F (1, 38) = 7.016, *p* = 0.012, partial η^2^ = 0.156]. A significant main effect of time was also found [F (1, 38) = 15.915, *p* < 0.001, partial η^2^ = 0.295]. Paired-sample *t*-tests indicated a significant pre–post difference in the SR group (*p* < 0.001), whereas no significant difference was observed in the NS group (*p* = 0.404). Between-group comparisons showed no significant difference at the pre-exercise time point (*p* = 0.781), while a significant between-group difference was observed at the post-exercise time point (*p* < 0.001). Regarding parasympathetic activity, a significant group × time interaction effect was observed [F (1, 38) = 14.105, *p* = 0.001, partial η^2^ = 0.271]. A significant main effect of time was also found [F (1, 38) = 31.103, *p* < 0.001, partial η^2^ = 0.450]. Paired-sample *t*-tests indicated a significant pre–post difference in the SR group (*p* < 0.001), whereas no significant difference was observed in the NS group (*p* = 0.209). Between-group comparisons showed no significant difference at the pre-exercise time point (*p* = 0.238) but a significant difference at the post-exercise time point (*p* = 0.001).

### 3.3. Comparison of HR and RPE Between the SR and NS Groups

**Figure 4:**
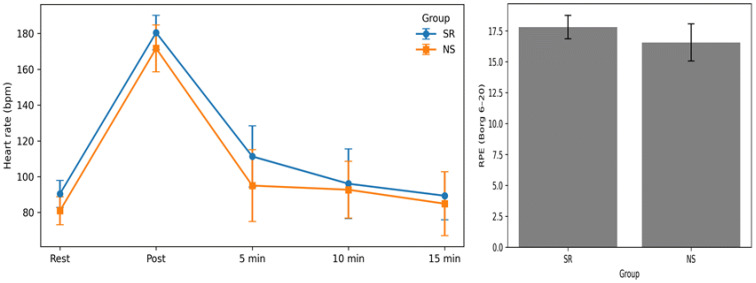
Heart rate and perceived exertion following high-intensity exercise under sleep restriction and normal sleep.

The resting HR differed significantly between the two groups [*t* (38) = 3.870, *p* < 0.001], with the SR group exhibiting higher values than the NS group (Table 4 and Figure 4). Similarly, the HR differed significantly between the two groups immediately after exercise [*t* (38) = 2.424, *p* = 0.020] and 5 min post-exercise [*t* (38) = 2.762, *p* = 0.009]. No significant between-group differences were found for HR at 10 min [*t* (38) = 0.596, *p* = 0.554] or 15 min post-exercise [*t* (38) = 0.890, *p* = 0.379]. RPE differed significantly between the two groups [*t* (38) = 3.142, *p* = 0.003], with the SR group exhibiting higher values than the NS group.

Additional analyses based on change scores (ΔHR from rest to immediately post-exercise) revealed no significant between-group difference in the magnitude of HR increase [*t* (38) = −0.151, *p* = 0.880]. These findings indicate that although absolute HR values were higher in the SR group, the relative exercise-induced increment was comparable between sleep conditions.

### 3.4. Comparison of Baseline Physical Fitness Factors Between the SR and NS Groups

No significant between-group difference was observed for muscular strength [*t* (38) = −0.975, *p* = 0.336] (Table 5). Likewise, flexibility [*t* (38) = −1.531, *p* = 0.134] and balance [*t* (38) = −1.661, *p* = 0.105] did not differ significantly between the two groups.

## 4. Discussion

### 4.1. The Central Nervous System’s Response to High-Intensity Exercise in the SR and NS Groups (TBR and SEF-90)

In this study, a significant increase in TBR and a significant decrease in SEF-90 was observed in the SR group post-exercise. These changes were primarily characterized by a significant group × time interaction effect. This interaction indicates differential temporal response patterns between groups rather than consistent between-group differences at a single post-exercise time point. However, no pre–post changes were observed in the NS group. Accordingly, the present findings should be interpreted with emphasis on change trajectories and interaction effects rather than absolute post-exercise group comparisons. These findings may reflect neural response patterns compatible with fatigue-related characteristics, although they do not constitute definitive evidence of central fatigue. Furthermore, the observed interaction effects for both TBR and SEF-90 were accompanied by moderate to large effect sizes (partial η^2^). This indicates that the impact of sleep restriction on the central nervous system’s response to high-intensity exercise is not only statistically significant but also practically meaningful.

The TBR is an EEG index that reflects arousal level, attentional regulation, and cognitive efficiency, with increased TBR generally reflecting accumulated central fatigue or reduced arousal. Previous studies have indicated that sleep deprivation is accompanied by increased theta-band activity and decreased alpha-band activity, and these EEG spectral alterations are associated with diminished arousal and impaired cognitive performance [26,27]. In particular, Snipes et al. [26] demonstrated that under conditions of elevated sleep pressure, increased theta activity can be concurrently observed with poor cognitive control and reduced performance. Accordingly, increased TBR may reflect not only sleepiness but also alterations in central nervous system functional regulation.

In this study, the finding that TBR increased significantly post-exercise only in the SR group suggests that, when arousal-maintenance resources were already reduced due to sleep restriction, the exercise stimulus may have further shifted EEG indices toward patterns compatible with central fatigue–related characteristics. The changes in SEF-90 observed in this study further support this interpretation. In particular, the significant group × time interaction observed for SEF-90 was accompanied by a relatively large partial η^2^, suggesting that the combination of sleep restriction and high-intensity exercise exerted a strong influence on alterations in the EEG frequency distribution. SEF-90 represents the frequency below which 90% of the cumulative power distribution of the EEG spectrum is contained. SEF-90 provides an index of spectral distribution rather than a direct mechanistic marker of specific neural processes; therefore, interpretations based on SEF-90 should be considered descriptive rather than mechanistic. Reduced SEF-90 indicates a relative shift in EEG energy toward lower-frequency bands. Previous studies have reported that, following sleep deprivation, resting-state EEG is characterized by decreased alpha-band power accompanied by an increased relative contribution of theta-band activity, which results in an overall slowing of the spectral distribution [27,28]. Such patterns of frequency redistribution have been interpreted as EEG slowing or spectral slowing phenomena associated with reduced central nervous system arousal and the accumulation of fatigue [29,30].

In this study, the finding that participants in the sleep restriction group exhibited decreased SEF-90 post-exercise may indicate that the exercise stimulus may have imposed an additional burden on an already compromised central nervous system, which may have been accompanied by a more pronounced shift in the EEG spectral distribution toward lower-frequency bands. This finding is consistent with previous experimental studies reporting that sleep deprivation leads to reductions in EEG power spectra and functional connectivity, accompanied by decreased arousal and vigilance levels [28,31]. In addition, it has been reported that EEG changes associated with central nervous system fatigue may manifest more sensitively when sleep restriction is combined with high-intensity or prolonged physical activity. Zhao et al. [14] reported that the performance of high-intensity endurance exercise is impaired under conditions of sleep deprivation (or partial sleep restriction). This is characterized by reduced exercise duration and increased maximal heart rate. Moreover, these performance decrements are accompanied by post-exercise alterations in theta-band power and patterns of functional connectivity. These findings suggest that, under conditions of insufficient sleep, exercise-induced stress may exacerbate neurophysiological burden, thereby increasing the likelihood of performance impairments.

While the observed EEG slowing and increased TBR are consistent with theoretical models of central fatigue, caution is warranted in attributing these changes solely to central fatigue mechanisms. EEG spectral indices are influenced by multiple physiological and cognitive factors, including baseline cortical arousal, sleep-related variability, and expectancy-related responses. Particularly in sleep manipulation studies, pre-existing differences in arousal state may partially contribute to post-exercise spectral changes. Therefore, the present findings should be interpreted within a multifactorial framework rather than as definitive or exclusive evidence of central fatigue.

Overall, these results suggest that under sleep restriction, high-intensity exercise may exert a greater influence on central nervous system frequency distribution and arousal-regulation mechanisms.

Our results demonstrated a significant between-group difference in SEF-90 at the pre-exercise time point; however, this difference was no longer evident at the post-exercise time point. These findings suggest that interpretation should emphasize pre–post changes and interaction effects rather than comparisons of absolute values alone. Under conditions of sleep restriction, when high-intensity exercise is imposed on a central nervous system with already reduced functional reserve, the observed pattern appears to be associated with a more distinct EEG response compatible with spectral slowing–related characteristics (i.e., increased TBR and decreased SEF-90), although the relatively short duration of the exercise stimulus warrants cautious interpretation. Importantly, this interpretation reflects differences in response trajectories across time rather than definitive between-group differences at the post-exercise time point. Taken together, our findings indicate that under sleep restriction, high-intensity exercise may be associated with alterations in central nervous system arousal regulation and information-processing efficiency, reflected by a relative shift in the EEG spectrum toward lower-frequency bands. Thus, under conditions of insufficient sleep, the same exercise stimulus may be accompanied by neural responses compatible with fatigue- or spectral slowing–related characteristics compared with normal sleep conditions; however, given that the present study relied on indirect EEG-derived indices, this interpretation should not be regarded as definitive evidence of central fatigue, but rather as indicative of altered neural response patterns within a broader multifactorial framework.

In addition, several methodological limitations should be explicitly acknowledged. In this study, EEG signal quality was monitored using manufacturer-recommended impedance thresholds and automated artifact-rejection algorithms embedded within the recording system. Although such procedures are widely used in applied neurophysiological research, reliance on embedded processing systems may impose certain constraints on methodological transparency and independent reproducibility. Furthermore, the EEG-derived indices analyzed in this study (TBR and SEF-90) should be regarded as composite measures reflecting overall spectral characteristics rather than direct mechanistic markers of specific neural processes. Accordingly, these indices may be limited with respect to independent validation and detailed physiological interpretation. Therefore, while the observed patterns are compatible with fatigue-related characteristics, they should be interpreted with caution and cannot be considered definitive physiological evidence of central fatigue.

### 4.2. The Autonomic Nervous System’s Response to High-Intensity Exercise in the SR and NS Groups

In this study, the SR group showed a significant reduction in the HRV index and parasympathetic activity and a significant increase in sympathetic activity post-exercise. These changes were primarily observed as significant group × time interaction effects. In contrast, the NS group did not show any significant pre–post changes. These findings suggest that the response of the autonomic nervous system to high-intensity exercise differs depending on sleep duration in healthy young adult women. Furthermore, the interaction effects observed for the HRV index as well as sympathetic and parasympathetic activity were accompanied by moderate to large effect sizes (partial η^2^), indicating that the impact of sleep restriction on autonomic regulatory responses to high-intensity exercise is not only statistically significant but also suggestive of meaningful differences in autonomic response patterns.

HRV is an index reflecting autonomic nervous system regulation based on beat-to-beat variations in one’s HR. Reduced HRV generally indicates sympathetic predominance or impaired parasympathetic modulation. Recent meta-analyses and experimental studies have consistently reported that, under conditions of sleep deprivation or sleep restriction, HRV is reduced not only at rest but also following stress or physical stimuli. Moreover, it is accompanied by increased sympathetic activity and decreased parasympathetic activity, reflecting an imbalance in autonomic response [32,33,34]. Such autonomic imbalance has been interpreted as reflecting less efficient physiological recovery regulation and increased cardiovascular burden. Although high-intensity exercise strongly activates the sympathetic nervous system, normal sleep enables rapid parasympathetic reactivation [35,36]. In contrast, sleep restriction or deprivation may delay or attenuate autonomic regulation during post-exercise recovery. Previous studies have shown that sleep deprivation or sleep restriction is associated with reduced HRV and a sustained predominance of sympathetic activity following physical stimuli, and such autonomic responses have been interpreted as reflecting altered or less efficient recovery regulation attributable to insufficient sleep [32,37,38].

In this study, the concurrent observation of reduced HRV, increased sympathetic activity, and decreased parasympathetic activity exclusively in the SR group post-high-intensity exercise suggests a less favorable pattern of autonomic recovery regulation under sleep restriction. Notably, the group × time interaction effects observed for these responses were accompanied by relatively large partial η^2^ values, indicating meaningful differences in response trajectories between groups rather than definitive impairment. These findings indicate that the autonomic regulatory demands imposed by exercise may vary according to sleep duration in healthy young adult women. Moreover, they suggest that the restoration of the sympathetic–parasympathetic balance may be relatively attenuated under conditions of insufficient sleep, although the short duration of the exercise protocol warrants cautious interpretation.

Accordingly, our findings concerning the autonomic nervous system suggest that under sleep restriction, high-intensity exercise may be associated with a pattern of autonomic response characterized by reduced HRV and relative sympathetic predominance. That is, when sleep restriction precedes high-intensity exercise, the regulatory burden on the autonomic nervous system may increase. Consequently, responses appear to shift toward a pattern characterized by relatively reduced parasympathetic reactivation during recovery. Taken together with the findings concerning the central nervous system, these results suggest that, in healthy young adult women, under conditions of insufficient sleep, high-intensity exercise may be accompanied by greater regulatory demands across the central–peripheral regulatory systems, although causal inferences should be interpreted cautiously.

In addition, methodological considerations specific to the PPG-derived autonomic indices should be acknowledged. In this study, autonomic parameters were derived from PPG signals using device-embedded algorithms. Although these indices are based on established time- and/or frequency-domain HRV components and have been used in applied research contexts, detailed information regarding the computational structure of these composite outputs is limited. Accordingly, these measures reflect autonomic regulatory patterns inferred from signal dynamics rather than direct recordings of sympathetic or parasympathetic neural activity. Therefore, while the observed autonomic responses are consistent with theoretical models of sleep-related autonomic imbalance, their physiological interpretation should be approached with appropriate caution.

### 4.3. HR, RPE, and Baseline Physical Fitness Factors in the SR and NS Groups

In this study, the SR group showed a significantly higher HR at rest, immediately post-exercise, and at 5 min post-exercise compared with the NS group, whereas no significant between-group differences were observed at 10 and 15 min post-exercise. Similarly, RPE was significantly higher in the SR group. These findings suggest that, in healthy women in their twenties, sleep restriction may be associated with greater cardiovascular and perceptual strain during high-intensity exercise and may also relate to a distinct early-phase pattern of post-exercise HR recovery. In particular, under sleep restriction, high-intensity exercise occurred with a higher resting HR; HR remained higher immediately after exercise and at the 5 min recovery time point, indicating differences in the early recovery profile between sleep conditions. Taken together, these findings suggest that sleep restriction may be associated with a slower early-phase HR recovery trend; however, because resting HR differed between groups, these results should be interpreted primarily as differences in recovery patterns rather than definitive evidence of impaired recovery.

Importantly, supplementary change-score analyses further indicated that the magnitude of HR increase from rest to immediately post-exercise did not significantly differ between groups, reinforcing the interpretation that the observed differences should be understood in light of baseline cardiovascular differences.

In addition, previous studies have reported that even short-term reductions in sleep duration can influence baseline cardiovascular responses, such as resting HR [39]. From this perspective, the higher resting HR observed in the SR group may reflect elevated cardiovascular arousal under sleep restriction prior to exercise; therefore, interpretations of post-exercise HR responses should consider this baseline difference.

In addition, Cincin et al. [40] reported that compared with normal sleep conditions, HR recovery at 30 s and 1 min post-high-intensity exercise is significantly attenuated under acute sleep deprivation. This finding directly demonstrates that insufficient sleep can impair autonomic re-regulation during the immediate and early phases of post-exercise recovery. Fullagar et al. [41] examined the effects of sleep reduction on exercise performance and physiological responses and found that sleep status influences physiological responses related to autonomic nervous system regulation. It has also been reported that sleep restriction can be accompanied by alterations in subjective exercise responses [39]. These findings explain the significantly higher RPE observed in the SR group.

From this perspective, it can be inferred that even under identical exercise conditions, subjective exercise burden may be perceived relatively higher under sleep restriction conditions than under normal sleep conditions. However, the ratings of perceived exertion may differ depending on participant characteristics, fitness level, and exercise modality. Therefore, the findings of this should be interpreted comprehensively alongside those of HR and autonomic nervous system indices. Moreover, given the presence of various individual characteristics, including sex, caution is warranted in their interpretation.

In this study, baseline physical fitness factors did not differ significantly between the two groups. This indicates that short-term differences in sleep duration are not immediately reflected in baseline physical fitness indices. In general, physical fitness is developed or modified through repeated training stimuli or long-term lifestyle changes, and it has been reported to exhibit relatively limited responsiveness to short-term physiological stressors such as acute sleep restriction. In particular, previous studies examining physical fitness factors under sleep restriction have reported that maximal strength, neuromuscular activation, and maximal aerobic capacity tend to remain preserved. They have also demonstrated that acute sleep restriction may not induce immediate changes in baseline physical fitness indices [42,43], aligning with our findings. In this study, resting HR, HR immediately after exercise and during the early recovery phase, as well as RPE, differed according to sleep duration. Taken together, these findings suggest that sleep restriction influences cardiovascular and subjective response patterns during high-intensity exercise, independent of changes in physical fitness. The inclusion of baseline physical fitness variables was intended to examine whether acute sleep restriction affects intrinsic physical capacity itself or primarily alters physiological and perceptual response patterns during exercise. That is, sleep duration may influence physiological and subjective exercise responses even in the absence of concomitant changes in physical fitness.

Although several interesting findings emerged from this study, several limitations should be acknowledged. First, sleep duration was self-reported and not objectively verified using actigraphy or polysomnography, although bedtime and wake-up times were confirmed via text messages to enhance compliance. Second, EEG- and PPG-derived indices were obtained using a portable device, which may limit measurement precision and interpretative scope compared with more comprehensive physiological assessment systems. Third, the sample consisted exclusively of healthy women in their twenties, and the study employed a single-session design; therefore, generalization to other age groups, sexes, or chronic sleep-restriction contexts should be made cautiously. Fourth, EEG recordings were restricted to the prefrontal region (Fp1 and Fp2) using a two-channel configuration, and activity in other cortical regions was not assessed; thus, conclusions regarding broader cortical integration should be interpreted cautiously. Although mixed-design ANOVA was employed to examine group × time interaction effects, baseline differences were observed in certain variables (e.g., SEF-90 and resting HR). Supplementary change-score analyses were conducted to assess the robustness of the interaction findings; however, future studies with larger samples may benefit from applying baseline-adjusted analytical approaches, such as ANCOVA or mixed-effects modeling incorporating baseline values as covariates, to further clarify the potential influence of initial group differences on post-exercise outcomes.

In addition, multiple outcome variables were analyzed without formal multiplicity correction. Although primary outcomes were predefined and interpretation emphasized interaction effects and effect sizes, an increased risk of Type I error cannot be entirely excluded. Furthermore, PPG-derived autonomic indices were generated using proprietary embedded algorithms. While based on time- and/or frequency-domain HRV components, the detailed computational structure and independent validation framework of these composite outputs are not fully disclosed. Accordingly, these measures should be interpreted as device-derived proxies of autonomic regulation rather than as direct physiological measurements of sympathetic or parasympathetic nerve activity.

Although participants in the menstrual phase were excluded, broader menstrual cycle phases were not controlled. Hormonal fluctuations may influence autonomic and central nervous system activity; however, random group assignment reduces, though does not eliminate, the likelihood of systematic imbalance. Endocrine biomarkers were not assessed to verify physiological stress responses, and future studies incorporating objective sleep measures, multi-channel EEG, hormonal markers, and longitudinal designs are warranted. Incorporating entropy-based approaches may further enhance understanding of central nervous system dynamics under combined sleep restriction and exercise stress.

## 5. Conclusions

Compared with the NS group, the SR group showed greater pre–post changes and a distinct response pattern across the exercise and early recovery period, including increased TBR, decreased SEF-90, altered HRV index, higher sympathetic activity, reduced parasympathetic activity, and elevated HR and RPE. These results suggest that, in healthy young adult women, high-intensity exercise performed under sleep restriction may be associated with relatively greater neurophysiological and autonomic regulatory demands during the immediate post-exercise period. In contrast, no between-group differences were observed in baseline physical fitness indices, indicating that short-term differences in sleep duration may be more sensitively reflected in immediate neurophysiological and perceptual responses following high-intensity exercise rather than in overall fitness level. Taken together, these findings suggest that, in healthy young adult women, sleep status prior to high-intensity exercise may be an important factor in modulating post-exercise neural responses and recovery patterns. However, these findings should be interpreted primarily in terms of change trajectories and group × time interactions, with consideration of baseline differences (e.g., SEF-90 and resting HR).

## Figures and Tables

**Table 1:** Characteristics of the participants.

Characteristic	Group		*p*
	SR Group (*n* = 20)	NS Group (*n* = 20)	
Age (years)	20.50 ± 1.10	20.85 ± 1.72	0.449
Height (cm)	163.52 ± 4.43	162.79 ± 3.88	0.586
Weight (kg)	54.90 ± 6.93	52.89 ± 4.72	0.598
Body Fat (%)	26.10 ± 5.76	27.06 ± 6.03	0.612
Total sleep duration (hours)	3.65 ± 1.08	7.90 ± 1.07	<0.001 ***

Values are presented as means ± standard deviations. Comparisons between groups were performed using independent *t*-tests. *** *p* < 0.001. SR, sleep restriction; NS, normal sleep.

**Table 2:** EEG-derived indices before and after high-intensity exercise in the SR and NS groups.

Variable	Group	Pre	Post	Source	F	*p*	Partial η^2^ [95% CI]
TBR (θ/β ratio)	SR NS	3.75 ± 1.29 3.95 ± 1.19	4.85 ± 1.13 4.20 ± 1.10	Group	0.522	0.474	0.014 [0.000, 0159]
				Time	10.565	0.002 **	0.218 [0.044, 0.404]
				Group × Time	4.188	0.048 *	0.099 [0.000, 0.288]
SEF-90 (Hz)	SR NS	26.93 ± 3.69 23.42 ± 5.44	20.92 ± 7.41 23.63 ± 4.69	Group	0.070	0.793	0.002 [0.000, 0.102]
				Time	11.714	0.001 **	0.236 [0.058, 0.425]
				Group × Time	13.472	0.001 **	0.262 [0.078, 0.451]

Values are presented as means ± standard deviations. A two-way mixed-design analysis of variance was used to examine the main effects of group and time, as well as the group × time interaction. Effect sizes are presented as partial eta squared (partial η^2^). * *p* < 0.05 and ** *p* < 0.01. CI, confidence interval; SR, sleep restriction; NS, normal sleep; TBR, theta-to-beta ratio; SEF-90, spectral edge frequency at 90%.

**Table 3:** PPG-derived autonomic nervous system indices before and after high-intensity exercise in the SR and NS groups.

Variable	Group	Pre	Post	Source	F	*p*	Partial η^2^ [95% CI]
HRV index	SR NS	12.80 ± 3.22 13.48 ± 2.92	9.90 ± 2.89 13.33 ± 3.33	Group	7.791	0.008 **	0.170 [0.013, 0.381]
				Time	5.574	0.023 *	0.128 [0.001, 0.335]
				Group × Time	4.513	0.040 *	0.106 [0.000, 0.310]
Sympathetic activity	SR NS	5.29 ± 0.97 5.38 ± 1.08	6.47 ± 0.57 5.61 ± 0.80	Group	3.046	0.089	0.074 [0.000, 0.268]
				Time	15.915	<0.001 ***	0.295 [0.080, 0.497]
				Group × Time	7.016	0.012 *	0.156 [0.008, 0.366]
Parasympathetic activity	SR NS	5.67 ± 0.45 5.51 ± 0.42	4.53 ± 0.62 5.28 ± 0.68	Group	5.281	0.027 *	0.122 [0.000, 0.328]
				Time	31.103	<0.001 ***	0.450 [0.225, 0.623]
				Group × Time	14.105	0.001 **	0.271 [0.067, 0.468]

Values are presented as means ± standard deviations. A two-way mixed-design analysis of variance was used to examine the main effects of group and time, as well as the group × time interaction. Effect sizes are presented as partial eta squared (partial η^2^). * *p* < 0.05, ** *p* < 0.01, and *** *p* < 0.001. CI, confidence interval; SR, sleep restriction; NS, normal sleep; HRV, heart rate variability.

**Table 4:** HR and RPE in the SR and NS groups.

Variable	SR Group (*n* = 20)	NS Group (*n* = 20)	*p*
Resting HR (bpm)	90.40 ± 7.56	80.95 ± 7.87	<0.001 ***
Post-exercise HR (bpm)	180.50 ± 9.62	171.7 ± 13.07	0.020 *
HR at 5 min post-exercise (bpm)	111.30 ± 17.15	95.00 ± 20.05	0.009 **
HR at 10 min post-exercise (bpm)	96.05 ± 19.47	92.70 ± 15.87	0.554
HR at 15 min post-exercise (bpm)	89.35 ± 13.46	84.90 ± 17.84	0.379
RPE	17.80 ± 0.95	16.55 ± 1.50	0.003 **

Values are presented as means ± standard deviations. Comparison between the two groups were performed using independent *t*-tests. * *p* < 0.05, ** *p* < 0.01, and *** *p* < 0.001. SR, sleep restriction; NS, normal sleep; HR, heart rate; RPE, rating of perceived exertion.

**Table 5:** Baseline physical fitness factors in the SR and NS groups.

Variable	SR Group (*n* = 20)	NS Group (*n* = 20)	*p*
Muscular strength (kg)	21.85 ± 4.32	23.07 ± 3.63	0.336
Flexibility (cm)	9.19 ± 10.16	13.60 ± 7.91	0.134
Balance (seconds)	28.02 ± 20.19	37.65 ± 16.30	0.105

Values are presented as means ± standard deviations. Comparison between the two groups were performed using independent *t*-tests. SR, sleep restriction; NS, normal sleep.

## Data Availability

The data presented in this study are available on request from the corresponding author due to ethical restrictions and the inclusion of sensitive participant information.

## References

[1] Alger S.E., Chambers A.M., Cunningham T., Payne J.D. (2015). The role of sleep in human declarative memory consolidation. Sleep, Neuronal Plasticity and Brain Function.

[2] Tempesta D., Socci V., De Gennaro L., Ferrara M. (2018). Sleep and emotional processing. Sleep Med. Rev..

[3] Walker M.P. (2009). The role of sleep in cognition and emotion. Ann. N. Y. Acad. Sci..

[4] Nikolic A., Bukurov B., Kocic I., Vukovic M., Ladjevic N., Vrhovac M., Sipetic S. (2023). Smartphone addiction, sleep quality, depression, anxiety, and stress among medical students. Front. Public Health.

[5] Zhu W., Liu J., Lou H., Mu F., Li B. (2024). Influence of smartphone addiction on sleep quality of college students: The regulatory effect of physical exercise behavior. PLoS ONE.

[6] Gordji-Nejad A., Matusch A., Kleedörfer S., Jayeshkumar Patel H., Drzezga A., Elmenhorst D., Bauer A. (2024). Single dose creatine improves cognitive performance and induces changes in cerebral high energy phosphates during sleep deprivation. Sci. Rep..

[7] Nieuwenhuys A., Wadsley C.G., Sullivan R., Cirillo J., Byblow W.D. (2025). Tired and out of control? Effects of total and partial sleep deprivation on response inhibition under threat and no-threat conditions. Sleep.

[8] Khan M.A., Al-Jahdali H. (2023). The consequences of sleep deprivation on cognitive performance. Neurosci. J..

[9] Li J., Cao Y., Ou S., Jiang T., Wang L., Ma N. (2024). The effect of total sleep deprivation on working memory: Evidence from diffusion model. Sleep.

[10] Messa R.M., Benfica M.A., Ribeiro L.F., Williams C.M., Davidson S.R., Alves E.S. (2024). The effect of total sleep deprivation on autonomic nervous system and cortisol responses to acute stressors in healthy individuals: A systematic review. Psychoneuroendocrinology.

[11] Ren Z., Mao X., Zhang Z., Wang W. (2025). The impact of sleep deprivation on cognitive function in healthy adults: Insights from auditory P300 and reaction time analysis. Front. Neurosci..

[12] Charest J., Grandner M.A. (2022). Sleep and athletic performance: Impacts on physical performance, mental performance, injury risk and recovery, and mental health: An update. Sleep Med. Clin..

[13] Roberts S.S., Teo W.P., Aisbett B., Warmington S.A. (2019). Effects of total sleep deprivation on endurance cycling performance and heart rate indices used for monitoring athlete readiness. J. Sports Sci..

[14] Zhao S., Alhumaid M.M., Li H., Wei X., Chen S.S.C., Jiang H., Qin H. (2025). Exploring the effects of sleep deprivation on physical performance: An EEG study in the context of high-intensity endurance. Sports Med.-Open.

[15] Kong Y., Yu B., Guan G., Wang Y., He H. (2025). Effects of sleep deprivation on sports performance and perceived exertion in athletes and non-athletes: A systematic review and meta-analysis. Front. Physiol..

[16] Temesi J., Arnal P.J., Davranche K., Bonnefoy R., Levy P., Verges S., Millet G.Y. (2013). Does central fatigue explain reduced cycling after complete sleep deprivation. Med. Sci. Sports Exerc..

[17] Martínez Vásquez D.A., Posada-Quintero H.F., Rivera Pinzón D.M. (2023). Mutual information between EDA and EEG in multiple cognitive tasks and sleep deprivation conditions. Behav. Sci..

[18] Banks S., Dinges D.F. (2007). Behavioral and physiological consequences of sleep restriction. J. Clin. Sleep Med..

[19] Mao T., Chai Y., Guo B., Quan P., Rao H. (2023). Sleep architecture and sleep EEG alterations are associated with impaired cognition under sleep restriction. Nat. Sci. Sleep.

[20] Van Dongen H.P., Maislin G., Mullington J.M., Dinges D.F. (2003). The cumulative cost of additional wakefulness: Dose-response effects on neurobehavioral functions and sleep physiology from chronic sleep restriction and total sleep deprivation. Sleep.

[21] Glavin E.E., Matthew J., Spaeth A.M. (2022). Gender differences in the relationship between exercise, sleep, and mood in young adults. Health Educ. Behav..

[22] Hajali V., Andersen M.L., Negah S.S., Sheibani V. (2019). Sex differences in sleep and sleep loss-induced cognitive deficits: The influence of gonadal hormones. Horm. Behav..

[23] Hoddes E., Zarcone V., Smythe H., Phillips R., Dement W.C. (1973). Quantification of sleepiness: A new approach. Psychophysiology.

[24] Shahid A., Wilkinson K., Marcu S., Shapiro C.M. (2012). Stanford Sleepiness Scale (SSS). STOP, THAT and One Hundred Other Sleep Scales.

[25] Borg G.A. (1982). Psychophysical bases of perceived exertion. Med. Sci. Sports Exerc..

[26] Snipes S., Krugliakova E., Meier E., Huber R. (2022). The theta paradox: 4-8 Hz EEG oscillations reflect both sleep pressure and cognitive control. J. Neurosci..

[27] Wu J., Zhou Q., Li J., Chen Y., Shao S., Xiao Y. (2021). Decreased resting-state alpha-band activation and functional connectivity after sleep deprivation. Sci. Rep..

[28] Lian J., Xu L., Song T., Peng Z., Zhang Z., An X., Shao Y. (2023). Reduced resting-state EEG power spectra and functional connectivity after 24 and 36 hours of sleep deprivation. Brain Sci..

[29] Corsi-Cabrera M., Ramos J., Arce C., Guevara M.A., Ponce-de León M., Lorenzo I. (1992). Changes in the waking EEG as a consequence of sleep and sleep deprivation. Sleep.

[30] Strijkstra A.M., Beersma D.G., Drayer B., Halbesma N., Daan S. (2003). Subjective sleepiness correlates negatively with global alpha (8–12 Hz) and positively with central frontal theta (4–8 Hz) frequencies in the human resting awake electroencephalogram. Neurosci. Lett..

[31] An X., Lian J., Xu L., Peng Z., Chen S., Cheng M.Y., Shao Y. (2024). Changes in electroencephalography microstates are associated with reduced levels of vigilance after sleep deprivation. Brain Res..

[32] Bourdillon N., Jeanneret F., Nilchian M., Albertoni P., Ha P., Millet G.P. (2021). Sleep deprivation deteriorates heart rate variability and photoplethysmography. Front. Neurosci..

[33] Schlagintweit J., Laharnar N., Glos M., Zemann M., Demin A.V., Lederer K., Fietze I. (2023). Effects of sleep fragmentation and partial sleep restriction on heart rate variability during night. Sci. Rep..

[34] Zhang S., Niu X., Ma J., Wei X., Zhang J., Du W. (2025). Effects of sleep deprivation on heart rate variability: A systematic review and meta-analysis. Front. Neurol..

[35] Dupuy A., Birat A., Maurelli O., Garnier Y.M., Blazevich A.J., Rance M., Ratel S. (2022). Post-exercise heart rate recovery and parasympathetic reactivation are comparable between prepubertal boys and well-trained adult male endurance athletes. Eur. J. Appl. Physiol..

[36] Ndongo J.M., Lele E.C.B., Guessogo W.R., Mbian W.M., Ayina C.N.A., Guyot J., Assomo-Ndemba P.B. (2023). Post-exercise heart rate variability recovery after 800-m endurance run load among Cameroonian adolescent’s males. Sports Med. Health Sci..

[37] Meerlo P., Sgoifo A., Suchecki D. (2008). Restricted and disrupted sleep: Effects on autonomic function, neuroendocrine stress systems and stress responsivity. Sleep Med. Rev..

[38] Tobaldini E., Costantino G., Solbiati M., Cogliati C., Kara T., Nobili L., Montano N. (2017). Sleep, sleep deprivation, autonomic nervous system and cardiovascular diseases. Neurosci. Biobehav. Rev..

[39] Dupuis G.L., Kaveney J., Lessard E., Wilson B., McGinnis G.R., Perkins R.K. (2025). Effects of short-term sleep reduction and aerobic exercise on metabolism and inflammation in healthy adults. Curr. Res. Physiol..

[40] Cincin A., Sari I., Oğuz M., Sert S., Bozbay M., Ataş H., Basaran Y. (2015). Effect of acute sleep deprivation on heart rate recovery in healthy young adults. Sleep Breath..

[41] Fullagar H.H., Skorski S., Duffield R., Hammes D., Coutts A.J., Meyer T. (2015). Sleep and athletic performance: The effects of sleep loss on exercise performance, and physiological and cognitive responses to exercise. Sports Med..

[42] Craven J., McCartney D., Desbrow B., Sabapathy S., Bellinger P., Roberts L., Irwin C. (2022). Effects of acute sleep loss on physical performance: A systematic and meta-analytical review. Sports Med..

[43] Vaara J.P., Oksanen H., Kyröläinen H., Virmavirta M., Koski H., Finni T. (2018). 60-hour sleep deprivation affects submaximal but not maximal physical performance. Front. Physiol..

